# Provider perception of cardiopulmonary quality in the simulated context

**DOI:** 10.1186/2197-425X-3-S1-A748

**Published:** 2015-10-01

**Authors:** G Gonzi, A Vezzani, F Corradi, A D'Errico, G Noto, G Artioli, P Ghirardi, L Bonfanti, A Capecchi

**Affiliations:** Cardiology, University Hospital of Parma, Parma, Italy; Cardiac Surgery ICU, University Hospital of Parma, Parma, Italy; Intensice Care Unit, E.O. Ospedali Galiera, Genova, Italy; Social Welfare Service, University Hospital of Parma, Parma, Italy; Emergency Department, University Hospital of Parma, Parma, Italy; Cardiology, Hospital of Bentivoglio, Bologna, Italy

## Introduction

The concept of self-assessment is a central mechanism in human behavioural change and should lead to desirable practice patterns. Few studies have investigated the relationship between physicians' perception of their ability to perform cardiopulmonary resuscitation (CPR) and the actual quality of the same.

## Objectives

The aim of this study was to investigate the relationships between the physiological and psychosocial variables of cardiac resuscitation in order to improve the involvement and motivation of professionals during training courses.

## Methods

During 2012, 314 medical staff of the Parma University Hospital were trained in basic life support defibrillation (BLSD). Before starting the course, the participants were randomly selected to create teams of two people working in the same department to take part in a simulation reproducing the first five minutes of a cardiac arrest in a medical or surgical department of our hospital before the intervention of the hospital emergency team. Before and after the simulation, each participant was asked to answer a self-efficacy questionnaire concerning the management of cardiac arrest using a 10-point scale. During the simulation, the time to activate the emergency response system, hands-on time, time to defibrillation, the number of compressions, and the percentage of correct compressions were recorded.

## Results

The time to activate the emergency response system was 70.5 ± 78.8 seconds; the system was not activated by 55 teams. The time to defibrillation was 148.6 ± 58.4 seconds; the defibrillator was used within 120 seconds by 44 teams, and was not used at all by 36 (22.9%). Average hands-on time was 166.20 ± 62.9 seconds. The mean number of compressions was 216.22 ± 115.57, 9.97 ± 21.23% of which were satisfactory.

Pre-simulation levels of self-efficacy of < 5 were declared by 36.5% of the participants, a level of 5 by 24.5%, and levels of 6-10 by 38.4%. After the simulation, the levels were unchanged in 38.3%, higher in 30.5%, and lower in 31.2%. There were no significant correlations between pre-simulation self-efficacy levels and actual performance; after the simulation, the correlations were closer.

## Conclusions

The medical staff declared individual perceptions of good levels of efficacy in managing a simulated cardiac arrest, but this did not match their actual skills. Still open questions are whether and how this psychosocial variable plays a role in the quality of CPR, and whether knowing their limited capacity to manage a cardiac arrest can encourage medical staff to undertake BLSD retraining.
